# Successful percutaneous closure of an iatrogenic right ventricular perforation utilizing an Angio-Seal device: a case report

**DOI:** 10.1093/ehjcr/ytaf128

**Published:** 2025-03-14

**Authors:** Abdulrahman Arabi, Amr A Ashour, Mawahib El Hassan, Bassam Shoman, Abdulwahid Al Mulla

**Affiliations:** Cardiology Department, Heart Hospital, Hamad Medical Corporation, Doha, Qatar; Department of Medical Education, Hamad Medical Corporation, Doha, Qatar; Cardiology Department, Heart Hospital, Hamad Medical Corporation, Doha, Qatar; Anesthesia Department, Heart Hospital, Hamad Medical Corporation, Doha, Qatar; Cardiothoracic Surgery, Heart Hospital, Hamad Medical Corporation, Doha, Qatar

**Keywords:** Right ventricular perforation, Pericardiocentesis, Angio-Seal, Pericardial effusion, Tamponade, Case report

## Abstract

**Background:**

Pericardiocentesis, a procedure with potential life-saving implications, can lead to complications such as cardiac perforation. Emergency open cardiac surgery, the conventional approach for addressing iatrogenic or traumatic cardiac perforations, is associated with unfavourable outcomes in high-risk patients. This report explores a case of accidental iatrogenic right ventricular (RV) puncture, which was effectively managed using a percutaneous closure device.

**Case summary:**

A 42-year-old male was hospitalized due to a persistent cough, dyspnoea, and fever, raising suspicion of tuberculosis. He was found to have a large pericardial effusion with signs of impending cardiac tamponade. During an attempted pericardiocentesis at an outside facility, his right ventricle was inadvertently perforated by a catheter. He was transferred to a specialized hospital to be assessed by a cardiac surgeon, where the heart team performed a percutaneous closure of the perforation using an Angio-Seal device, which was successful. A subsequent pericardiectomy was performed to address the effusion and confirm the absence of tuberculosis or malignancy. The patient recovered well, and follow-up imaging showed no worsening of the pericardial effusion. He was discharged in stable condition.

**Discussion:**

Pericardiocentesis, while crucial for treating large pericardial effusions, carries a small risk of complications such as ventricular perforation. Although surgery is a cornerstone in management, the Angio-Seal device has been used successfully in some cases to repair these tears. In this case, an Angio-Seal device effectively closed a RV rupture, with no further complication seen in follow-up exams. This suggests Angio-Seal as a potentially useful, less invasive option for treating such complications.

**Learning points**

- To highlight an alternative management for an iatrogenic right ventricular puncture, which is a percutaneous angio-seal closure.
- To highlight the critical role of a collaborative heart team (cardiothoracic surgeon, interventional cardiologist, anaesthetist, and intensivist) in managing complex iatrogenic complications, ensuring rapid decision-making and tailored interventions.

## Introduction

Cardiac tamponade represents a critical medical condition characterized by the rapid or gradual compression of cardiac chambers, leading to haemodynamic instability. Pericardiocentesis is a pivotal and life-saving intervention for both diagnosing and managing substantial pericardial effusions and cardiac tamponade.^1^ This procedural approach involves utilizing a needle and a small catheter to drain accumulated fluid from the pericardial sac surrounding the heart.^2^

Large-scale observational studies employing echocardiography-guided pericardiocentesis in non-emergent cases reveal a major complication rate of <2%.^3^ Myocardial puncture stands out as one of the most serious and immediate mechanical complications associated with pericardiocentesis.^3^ While right ventricular (RV) perforation is a rare yet life-threatening consequence of pericardiocentesis, conventional treatment typically involves surgical intervention.^4^ However, noteworthy is the reported success of employing percutaneous device closure for RV perforation as a viable alternative, demonstrating lower morbidity rates.^5^

## Summary figure

**Figure:**
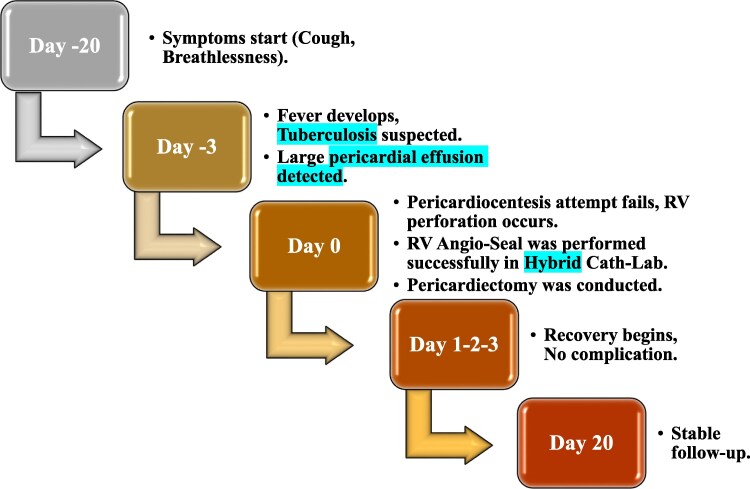


## Case presentation

A 42-year-old male of Indian descent, known to have diabetes mellitus and skin psoriasis, was hospitalized at a peripheral medical facility due to a persistent cough and 20 days of dyspnoea, accompanied by a 3-day history of fever, raising suspicion of tuberculosis. Upon clinical evaluation, he exhibited mild dyspnoea, maintaining a blood pressure of 117/75 mmHg, a heart rate of 110 beats per minute, and a temperature of 37.7°C. Physical examination also revealed jugular venous distention, tachycardia, and muffled heart sounds, which raised concern for impending tamponade, despite the stable blood pressure.

**Figure 1:**
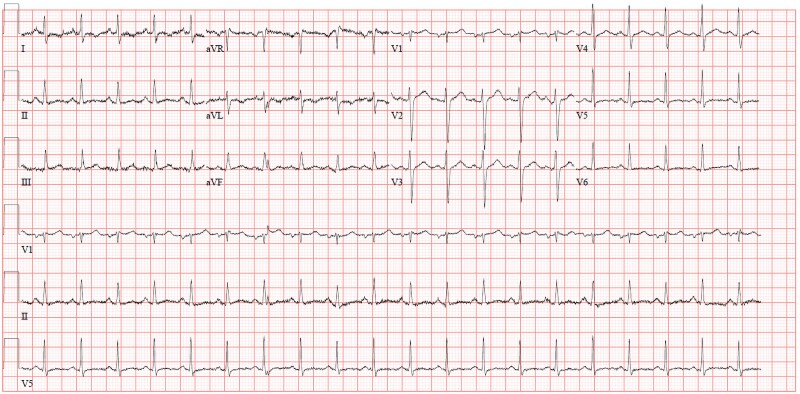
ECG.

Laboratory testing was significant for mild microcytic hypochromic anaemia with a haemoglobin of 11.6 g/dL (normal range: 13–17 g/dL for males). Additionally, C-reactive protein was elevated at 47 mg/L (normal range: <5 mg/L). The rest of the blood counts, complete metabolic panel, and cardiac enzymes were unremarkable. ECG illustrated sinus tachycardia (*Figure 1*), while chest X-ray was only significant for cardiomegaly. Echocardiography showed a large circumferential pericardial effusion, measuring 2.9 cm, with right atrial and right ventricular collapse indicating impending cardiac tamponade.

**Figure 2:**
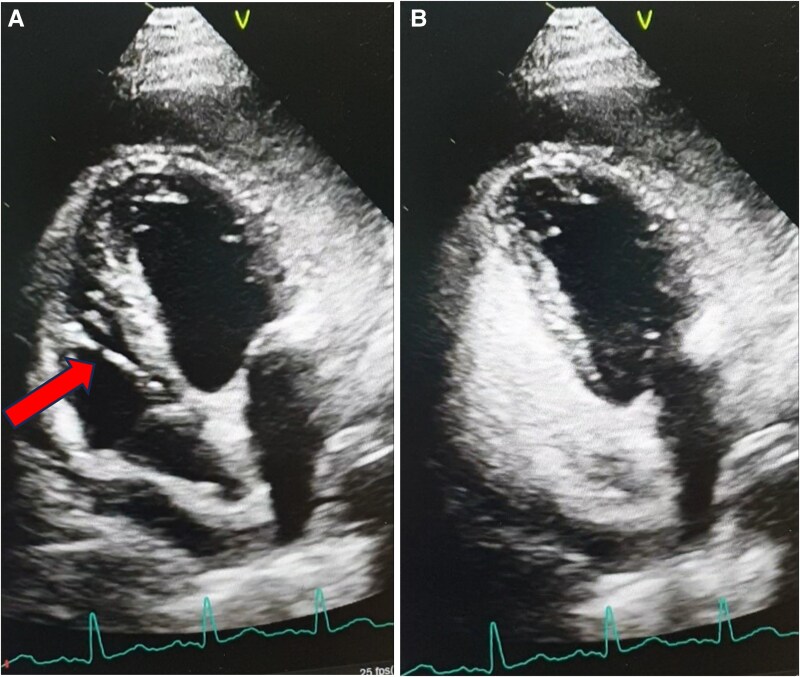
(*A*) Right ventricle perforation evidenced by the presence of pigtail catheter which was seen by echocardiography inside right ventricle. (*B*) Echocardiography image shows that agitated saline is seen inside the right-side cavity of the heart.

As haemodynamic stability can often be maintained in the early stages of tamponade, and this can rapidly progress to a more severe state, the primary medical team attempted pericardiocentesis, which, unfortunately, resulted in right ventricular perforation, as evidenced by the presence of a pigtail catheter visualized by echocardiography within the right ventricle, extending to the right atrium (*Figure 2A*). Agitated saline was observed within the right heart (*Figure 2B*). The pigtail catheter (6-French catheter) was retained in position. The patient remained stable on a modest dose of noradrenaline (0.08 mcg/kg/minute).

The patient was transferred to a hospital with cardiac surgery for surgical repair of right RV perforation. Upon arrival, the heart team (cardiothoracic surgeon, interventional cardiologist, cardiac anaesthetist, and cardiac intensivist) reviewed the case and decided to proceed with percutaneous closure in the hybrid cardiac catheterization laboratory with surgical backup for immediate surgical repair in case of percutaneous closure failure. This management decision was made in consultation with the heart team to address the immediate risk of tamponade and to seal the RV perforation, as it was crucial to ensure the immediate stability of the patient, which percutaneous closure helps to achieve.

Under fluoroscopic and transthoracic echocardiographic guidance, a guidewire was carefully advanced through the pericardial access sheath into the right ventricular cavity. A 6-French Angio-Seal device was subsequently deployed over the wire at the site of perforation, ensuring precise placement. Real-time imaging confirmed successful sealing of the defect, with no evidence of residual leakage. Immediate post-procedural echocardiography demonstrated a reduced volume of pericardial effusion, with no new signs of tamponade. The patient remained haemodynamically stable throughout the procedure, with no complications observed.

**Figure 3:**
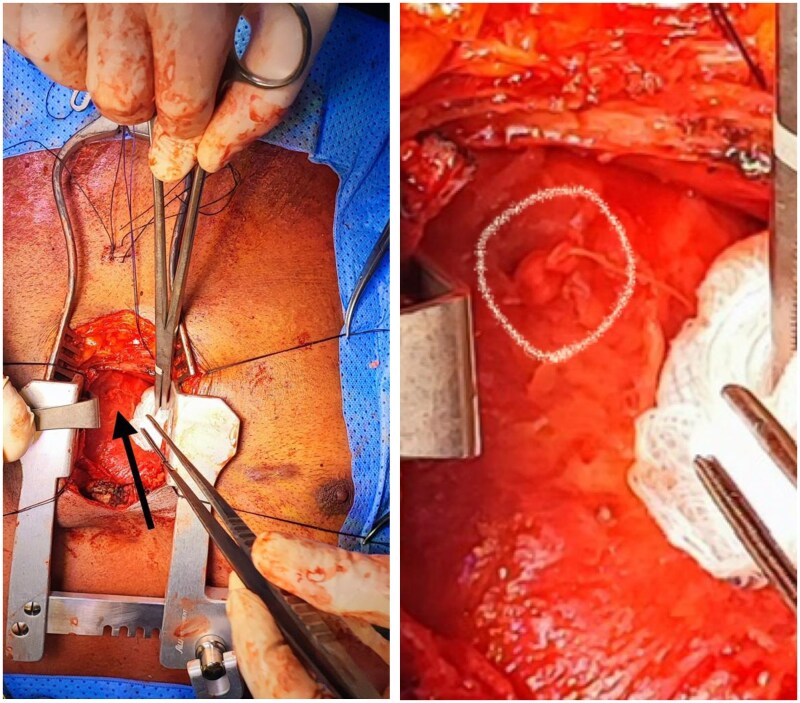
Closure of the iatrogenic right ventricle perforation using a 6 French Angio-Seal.

After the successful closure and due to the high suspicion of tuberculosis, a lower median sternotomy was undertaken with a partial pericardiectomy to drain the pericardial fluid, obtain tissue diagnosis, and mitigate the risk of constrictive pericarditis. The successful deployment of Angio-Seal was confirmed with visual exploration (*Figure 3*).

Analysis of the pericardial fluid revealed no bacterial growth. The serum autoimmune panel was unremarkable. Pericardial biopsy was negative for both granulomas and malignancy, suggesting a viral infection as a potential differential diagnosis. Subsequent follow-up echocardiography, performed 2 days later, revealed a moderate pericardial effusion, measuring 1.1 cm, with no signs of tamponade. The patient was discharged in stable condition, with close follow-up appointments scheduled at the clinic. Follow-up echocardiographic assessment at three weeks showed no discernible expansion in pericardial effusion. The patient remained asymptomatic and stable, including normal vital signs and no further clinical concerns.

## Discussion

Pericardiocentesis has long been a widely employed procedure for the diagnostic and therapeutic management of significant pericardial effusion, particularly in cases of cardiac tamponade, where it serves as a life-saving intervention. The 2015 ESC Guidelines for the diagnosis and management of pericardial diseases offer comprehensive recommendations, further reinforcing the established role of this procedure in clinical practice.^4^

Despite its routine administration under the guidance of transthoracic echocardiography or fluoroscopy, pericardiocentesis is not without inherent risks and potential complications. Large-scale observational studies employing echocardiography-guided pericardiocentesis in non-emergent cases reveal a major complication rate of <2%.^3^ In contrast, in emergent or complex cases, such as rescue pericardiocentesis for cardiac perforation, the overall major complication rate is documented at 3%, with iatrogenic ventricular perforation occurring in 1% of procedures.^6^

While surgical exploration, identification of the rupture site, and suture closure of the defect represent established and effective therapeutic modalities in the majority of patients, noteworthy instances have been reported wherein iatrogenic perforations were technically and successfully addressed using Angio-Seal devices.^7^

In the current case, the closure of the iatrogenic right ventricular free wall perforation was accomplished using a 6 French Angio-Seal device. Nevertheless, the patient necessitated surgical sternotomy for diagnostic biopsy purposes. Subsequent echocardiographic assessments performed over a 20-day period post-procedure revealed no discernible augmentation in pericardial effusion. Our observations suggest that the Angio-Seal vascular closure device may offer a promising and minimally invasive approach for managing this rare yet potentially serious complication arising from pericardiocentesis.

## Conclusion

In conclusion, based on the available literature and clinical evidence, percutaneous closure of inadvertent right ventricular perforation following pericardiocentesis may offer a feasible alternative to surgical intervention. While the evidence supporting its safety and efficacy is still emerging, this approach could represent a valuable addition to the range of interventions for managing this complication.

## Lead author biography


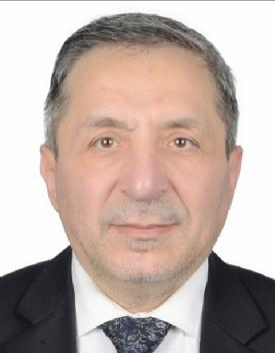


Abdulrahman Arabi, MD FACC. Graduated from Damascus University, Syria in 1994. Internal Medicine residency, cardiology and interventional cardiology fellowships: St John Hospital, Detroit, MI, USA, 1999–2006. 2006: interventional cardiology consultant at Henry Ford Hospital, Detroit, MI. 2008: appointed director of PPCI programme. 2010: joined HMC, Qatar. 2014: till now, deputy chief of cardiology and director of the Cath lab. Special interest: cardiogenic shock and invasive haemodynamics.

## Data Availability

No new data were generated or analysed in support of this research.

## Notes

**Consent:** The authors confirm that written consent has been obtained from the patient for the publication of this case in accordance with COPE guidelines.

**Funding:** This article will be funded by Qatar National Library.

**Ethics approval:** Medical Corporation Medical Research Center. This research was conducted ethically in accordance with the World Medical Association Declaration of Helsinki. The subject in this paper has given their informed consent to publish their case.
